# Aspects of Nickel, Cobalt and Lithium, the Three Key Elements for Li-Ion Batteries: An Overview on Resources, Demands, and Production

**DOI:** 10.3390/ma17174389

**Published:** 2024-09-05

**Authors:** Paul Kalungi, Zhuo Yao, Hong Huang

**Affiliations:** 1 Department of Mechanical and Materials Engineering, Wright State University, Dayton, OH 45435, USA; kalungi.2@wright.edu; 2 Department of Materials Science and Engineering, University of Science and Technology Liaoning, Anshan 114051, China; yaozhuo1986@163.com

**Keywords:** lithium, nickel, cobalt, metallurgical, recovery, extraction

## Abstract

With the booming of renewable clean energies towards reducing carbon emission, demands for lithium-ion batteries (LIBs) in applications to transportation vehicles and power stations are increasing exponentially. As a consequence, great pressures have been posed on the technological development and production of valuable elements key to LIBs, in addition to concerns about depletion of natural resources, environmental impacts, and management of waste batteries. In this paper, we compile recent information on lithium, nickel, and cobalt, the three most crucial elements utilized in LIBs, in terms of demands, current identified terrestrial resources, extraction technologies from primary natural resources and waste. Most nickel and cobalt are currently produced from high-grade sulfide ores via a pyrometallurgical approach. Increased demands have stimulated production of Ni and Co from low-grade laterites, which is commonly performed through the hydrometallurgical process. Most lithium exists in brines and is extracted via evaporation–precipitation in common industrial practice. It is noteworthy that at present, the pyrometallurgical process is energy-intensive and polluting in terms of gas emissions. Hydrometallurgical processes utilize large amounts of alkaline or acidic media in combination with reducing agents, generating hazardous waste streams. Traditional evaporation–precipitation consumes time, water, and land. Extraction of these elements from deep seas and recycling from waste are emerging as technologies. Advanced energy-saving and environmentally friendly processes are under extensive research and development and are crucial in the process of renewable clean energy implementation.

## 1. Introduction

**Figure 1:**
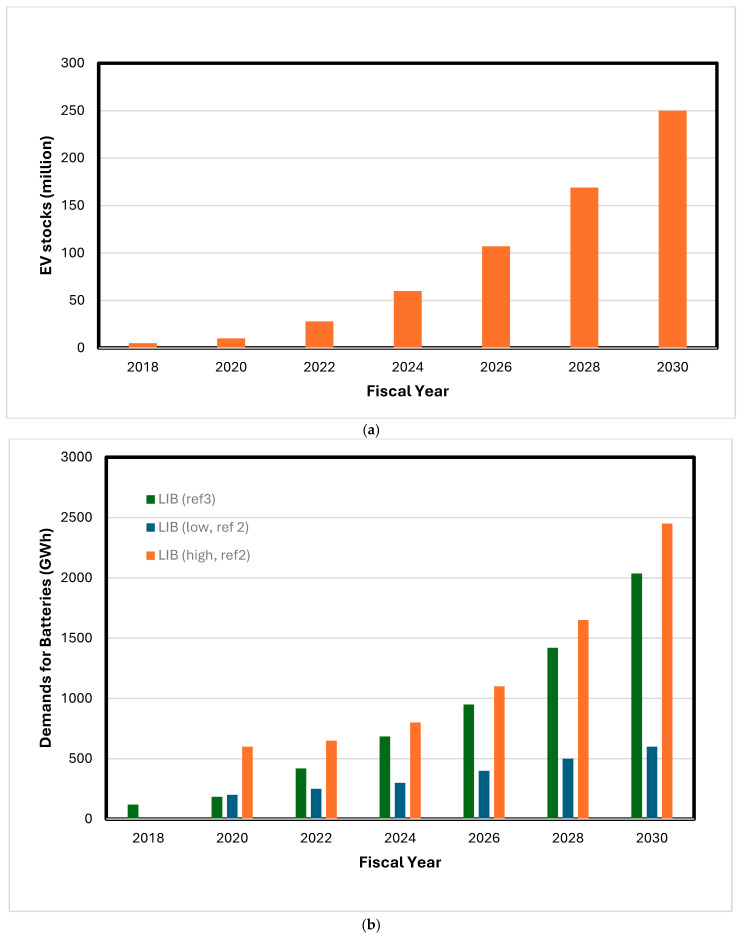
(**a**) Global EV (including plug-in EVs and battery EVs) stocks by fiscal year (replotted from refs. [1,2]); (**b**) global demands for LIB (replotted from refs. [3,4]).

Electric vehicles (EVs) and renewable clean energy (RCE) power grid systems are booming at an accelerating pace with rapid technological progress and governmental policy stimuli. Global EV sales grew from 7.5 million in 2019 to over 40 million in 2023, of which 70% are battery electric vehicles (BEVs). EV stocks are projected to reach 250 million by 2030 (see Figure 1a) [1,2]. The increased demands for EVs result in a proportionate increase in battery demand and production (see Figure 1b) [3,4]. Implementation of wind or solar systems for green energy generation requires stationary energy storage systems (ESS). The global ESS market in terms of power was 222.8 GW in 2022 and is expected to reach 512.4 GW by 2030 based on a compound annual growth rate (CAGR) of 11.6% from 2023 to 2030 [5]. Battery energy storage systems (BESSs) are projected to double in less than 10 years and reach between 120 billion USD and 150 billion USD in market value by 2030 [6].

**Figure 2:**
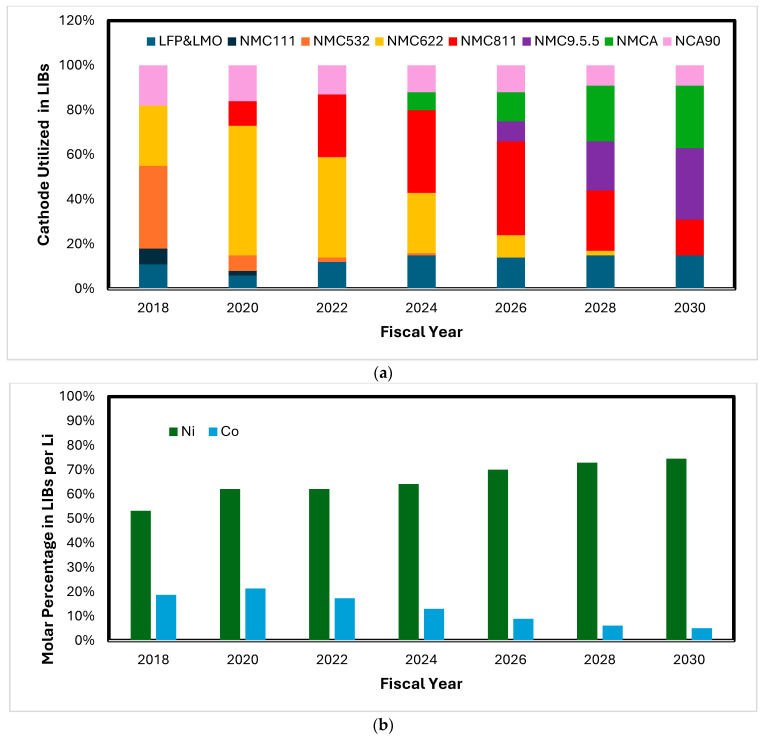
(**a**) Various cathode materials and their percentage utilized in LIBs (replotted from refs. [10,11]); (**b**) molar fraction of Ni and Co per Li utilized in LIBs, calculated based on the cathode composition and the percentage of each cathode utilized.

Lithium-ion batteries (LIBs) offer high energy density and long cycle life comparing with other electrochemical energy sources, making them predominantly applied in EVs and BESSs at present and in the near future. A LIB is composed of four basic components: a cathode, an anode, an electrolyte w/o separator, and an outer casing [7]. The cathode is one of the most critical materials determining LIB performance. In a typical battery setting, the cathode contributes 25–30% of a battery weight, in which 80–85% is from the active lithium transition metal oxide cathode [8]. Active cathode materials in commercial LIBs can be LCO (LiCoO_2_), LFP (LiFePO_4_), NMC (LiNi_x_Mn_y_Co_z_O_2_), or NCA (LiNi_x_Co_y_Al_x_O_2_), in which NMC and NCA comprise various ratios of metals. In an NMC-based cathode, the mass compositions of each key elements are 1.2 to 2.0 wt% for Li, 2–8.5 wt% for Co, and 12–15 wt% for Ni, depending on the NMC chemistry. Each cathode’s chemistry grants it unique merits, and hence, different cathodes are preferred for different applications [9,10,11,12,13,14]. For instance, LFP is advantageous for its high performance and low cost, but it has a low gravimetric energy density. LCO is mostly used in consumer electronics for its technological maturity. With the skyrocketing price of Co, cathode materials are gradually transitioning to LiNiO_2_-based chemistry. NMC111 (Ni:Mn:Co 1:1:1, also referred to as NMC333) has a very high energy density, which is an ideal choice for a high-mileage EV. NMC811 (LiNi_0_._8_Mn_0_._1_Co_0_._1_O_2_) and NCA90 (LiNi_0_._9_Co_0_._05_Al_0_._05_O_2_) are being taken up rapidly for both their high energy density and their low cobalt content (see Figure 2a) [10,11]. According to the percentage of each type of cathodes utilized in commercial LIBs and their chemical compositions, we estimated Ni and Co molar fractions in the LIB consumption, normalized to Li in cathode consumption, which is shown in Figure 2b. It can be seen that Co consumption in commercial LIBs decreases from 20% to about 5%, while Ni consumption increases from 54% to 75%.

Great pressure has been placed on the technological development and production of valuable elements key to LIBs. On the one hand, concerns over the sustainability of these key elements have been raised. Assuming the increase trend of EVs and BSEEs persists within thirty years, demands for the key metallic elements utilized in LIBs will increase dramatically, i.e., lithium by a factor of 18–20, cobalt by 17–19, and nickel by 28–31 [15]. On the other hand, significant increases in waste LIBs are expected in the next decade. The waste LIB generation rate has been estimated based on EV sales, expected EV lifespan (8–10 years), annual lithium production, typical LIB masses, etc. About 1 MT of LIBs produced in 2016 would enter the waste stream in 2025, corresponding to 15, 50, and 150 kilo tons of Li, Co, and Ni, respectively. To address the potential resource depletion and waste accumulation, technological improvement in processing of these three key elements from land reserves to recycling them from waste has become imminent and crucial. 

**Figure 3:**
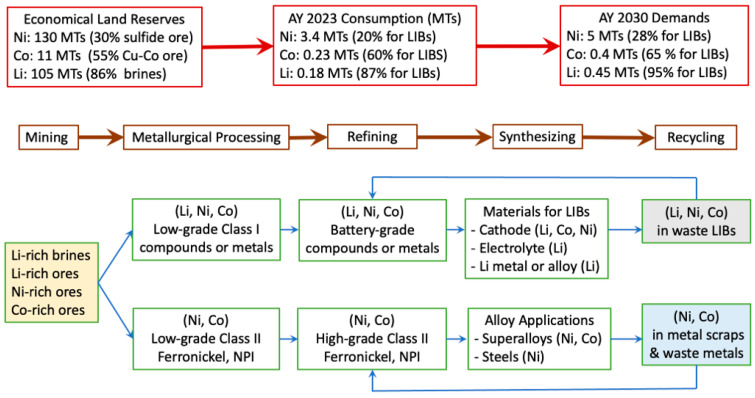
General flowcharts of consumption, processing, and footprints of the three key metals, i.e., Li, Ni, and Co.

Figure 3 shows a general flowchart of the consumption, processing, and footprints of Li, Co, and Ni, from natural reserves to end use. In general, extraction of these valuable elements, either from natural mineral ores or from secondary resources, is classified into pyrometallurgical and hydrometallurgical technologies. Pyrometallurgical processing is thermal treatment to target and extract valuable elements or compounds based on the difference in melting points, density, or other physical properties of common compounds like oxides or sulfates of various metals present in the natural ores. Pyrometallurgical processing generally involves roasting at intermediate temperatures (700–1000 °C) and smelting at high temperatures (1300–1600 °C) and hence is energy-intensive. In terms of hydrometallurgical processing, valuable metals are separated from other impurities, such as metals, in the form of solutions at low temperatures according to the differences in solubilities or other electrochemical properties. Hydrometallurgical processes include leaching, precipitation, and separation via solution extraction, which generally requires corrosive bases or acids and hence is not environmental-friendly. Concentration of lithium from brines can be realized via natural evaporation. However, this is time- and land-consuming. Mining products are usually at the intermediate-grade level (containing less than 50% valuable elements). For synthesizing battery-grade cathode or electrolyte materials, high-purity compounds of Li, Co, and Ni, in the form of acetate, carbonate, chloride, oxide, hydroxide, and sulfates, like cobalt sulfate (CoSO_4_), nickel sulfate (NiSO_4_), lithium hydroxide (LiOH·H_2_O), or lithium carbonate (Li_2_CO_3_), are indispensable. A wide range of processes, such as oxidation, leaching, precipitation, adsorption, ion exchange etc., have been adopted to remove impurities and to ensure the purity of the final compounds in refinery industries.

We recently conducted literature research on Li, Co, and Ni, the three crucial elements for LIBs. It was noticed that many data related to natural resources, demand, and production are widespread in business reports, and some are outdated. Sparse reviews found on mining production technologies are usually on specific elements or processing. By contrast, many review papers have emerged recently in line with recycling of LIBs [16,17,18,19,20,21,22,23,24,25,26]. In this paper, we aim to compile information on Li, Co, and Ni, based on the latest resources, in terms of the geographical resources of major minerals and commercial and state-of-the-art technologies in metallurgical production from mineral ores. These technologies can be modified and utilized for deep-sea mining and waste recycling. To limit the length of this manuscript, extraction of these three elements from the deep sea and recycling them from waste scraps and spent batteries are briefly summarized. Processing in terms of refining and synthesizing materials for LIBs is out of the scope of this paper.

## 2. Nickel

### 2.1. Demands and Land Reserves

Global nickel consumption in 2023 is nearly 3.4 million metric tons (MTs), of which about 20% is consumed for LIB cathode production. Annual nickel consumption for LIBs is estimated at 0.6–0.8 MTs in 2025. In 2030, nickel consumption will reach 5.0 MTs, of which 28% will be utilized for the cathodes of LIBs [2]. Although nickel is known to be the 24th most abundant metal, with more than 350 MTs in the earth’s crust, the identified economical land ores (above 1% Ni) are only 130 MTs at present. About 74% of nickel land reserves are concentrated in Indonesia (55 MTs), Australia (24 MTs), Brazil (16 MTs), Russia (8.3 MTs), and New Caledonia (7.1 MTs). 

Natural nickel minerals are categorized into magmatic sulfide and laterite deposits at a ratio of 3:7 [27,28,29,30]. Magmatic sulfide deposits form directly from molten rocks, in which the principal nickel-bearing mineral is (Ni,Fe)_9_S_8_ (pentlandite). Magmatic sulfide ore can have a nickel content of up to 8%. The type of laterite deposits varies depending on the local climatic conditions. Two main types of nickel-bearing laterite deposits are as follows: (1) limonite is basically iron oxyhydroxide goethite minerals incorporated into a certain amount of nickel. Limonite ore usually contains lower-grade Ni from 1.2% to 1.7% and high-content Fe up to 35%; (2) nickel-bearing saprolite is a silicate-based mineral. Smectite silicate clay (Al,Si)_3_O_4_ deposits usually occur in the middle or upper saprolite zone. Hydrous magnesium-nickel silicates, (Ni,Mg)_3_Si_2_O_5_(OH)_4_.6H_2_O, occur in the lower saprolite zone underneath the oxide laterite. Relative to limonite, saprolite laterite ores have a higher Ni grade in the range of 1.5–3% and lower Fe content (about 15%) but possible high Mg content. Apparently, high-grade magmatic sulfide ore is the most favorable among these three.

Nickel-based commercial products are classified into two categories. Class I comprises electrolytic nickel, powders, briquettes, carbonyl nickel, and nickel compounds, which are suitable for processing active cathode materials for LIBs. Class II is basically iron-bearing nickel including nickel pig iron (NPI, 3–5% nickel) and ferronickel (FeNi, 20–38% nickel), which are utilized for steel and superalloy production. At present, only 40% of total nickel mining outputs are Class I products.

### 2.2. Nickel Production from Land Ores

**Figure 4:**
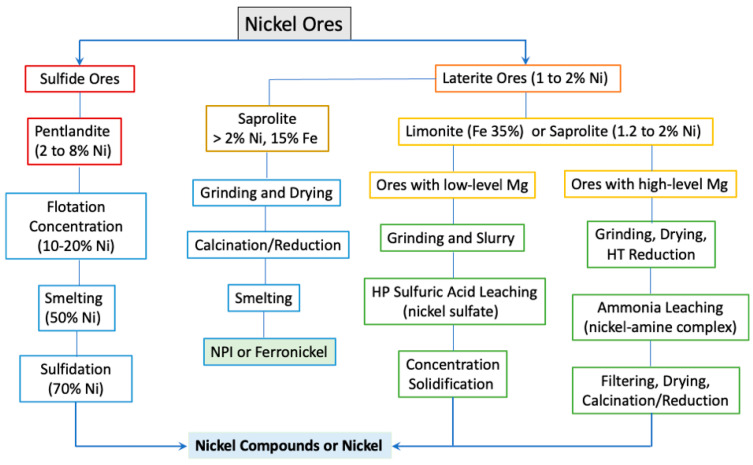
Typical flowchart of different processes for extracting Ni from different natural ores.

Choice of metallurgical method depends on the type of nickel ore. Figure 4 summarizes the typical flowchart of processing Ni from different natural ores. Sulfide ores and high-grade (>2% Ni) low-iron content lateritic ores are best suited to the pyrometallurgical process [31]. High-iron limonite or low-grade saprolite ore (Ni content 1.2% to 2%) is mainly processed via hydrometallurgy [32]. Limonite–saprolite blends and low-nickel saprolitic laterite are usually treated using a hybrid of pyro- and hydro-metallurgy. At present, over 60% of nickel is produced from sulfide ores. The increased demand for nickel has accelerated the development of producing nickel from lateritic deposits.

#### 2.2.1. Pre-Concentration

Due to the general low concentration of nickel in land ores, the pre-concentration step after mining is indispensable. This will reduce the requirements for energy and reagents and will increase the production rate. The first level of pre-concentration is crushing. Usually, several stages of crushing are undergone to maximize the separation of the ore minerals from the gangue (a coarse fraction containing ferric oxide, silicon dioxide, magnesium oxide, calcium oxide, aluminum oxide, etc.). The dry-crushed ore particulates can be mixed with water to create a slurry which is then fed into a series of grinders to produce powders of the required size. For effective extraction of nickel, laterites are required to be ground to a fine particle size (less than 40 μm). For low-grade and/or high-iron laterites, advanced pre-concentration techniques have been developed in the laboratory and tested at the pilot scale. These include gravity separation, magnetic separation, electrostatic separation, pre-roasting, etc. Although the advanced techniques have provided a certain degree of upgrading (see Table 1), it is still challenging to incorporate them into full plants [33] in consideration of capital and operating costs as well as the complex mineralogy of Ni laterite variations. 

The second level of pre-concentration is flotation. Fine ore powders are dispersed in water, forming a suspension solution. The water suspension is then treated with special collector-containing formulations to form a mineral froth, from which the hydrophilic phase is separated from the hydrophobic phase. Flotation is commonly used with sulfide minerals with collectors like xanthate, black medicine, and thiourethane [41,42,43,44]. Other additives such as pH adjuster, pulp dispersant, silicate mineral inhibitor, foaming agent, etc. are often added to increase flotation recovery efficiency [45,46,47,48,49]. Through this selection process, nickel concentration in solids increases to 10–20%. The floatability of ore particles is known to depend strongly on particle size. Recovery efficiency is maximised for particles in the range of 15 to 100 μm in size. Effective flotation from coarse particles is beneficial in terms of technical, economic, and sustainability aspects. Fluidized bed flotation can be promising and effective for coarse particle floatation [50].

Because low-grade laterite particles lack a specific Ni-rich phase on the particle surface to interact with collector molecules, effective flotation for laterite ore is still challenging and has not been implemented in present industries. Various flotation conditions have been investigated since 1973, but most results showed either minor upgrades or low recovery efficiency [51,52,53,54]. Various collectors have been tested, including hydroxamate (AM14), oleic acid, and cetyl trimethyl ammonium bromide (CTAB), just name a few. In a saprolite sample, nickel grade increased only 40% at about 70% recovery using a hydroxamate collector at pH 10 [54]. High-temperature roasting prior to flotation appears to be more effective. The roasting process was conducted in the presence of an oxidant, a reductant, or sulfur. The results showed oxidation or reduction was much less effective than sulfidation. Addition of sulfurous compounds significantly increased nickel grade and recovery with the highest recovery of 98% [55,56].

#### 2.2.2. Processing of Sulfide Ores

Currently, the main industrial process to extract nickel from high-grade sulfide ores is based on the pyrometallurgical method [31]. In general, dry sulfide concentrates after flotation are smelted at temperatures around 1300 °C in an electric roaster furnace or up to 1600 °C in a flash-smelting furnace. Flash-smelting is advantageous in terms of its short time and complete reaction. During this process, nickel and other metal sulfides melt as a eutectic liquid (matte), which can be separated from solid slag made up of impurities such as oxides and silicates. Ni content in the collected matte increases to around 50% Ni. The matte is then roasted in air at 500–700 °C, converting the nickel sulfide into nickel sulfate. By contrast, iron sulfide will be transformed into oxide. Nickel, as well as any Cu and Co present, is then hydrometallurgically extracted in the aqueous solution. The leaching rate of nickel is 92.1%, and 97% of nickel can be extracted after roasting [57,58]. The dried solid contains about 70% Ni. Further refining can produce high-purity class I nickel compounds or nickel metals.

Pyrometallurgical processing suffers from the drawbacks of high energy costs, serious nickel losses, inefficiency with low-grade sulfide ore, and high emissions of waste gases. A pyrometallurgical pretreatment followed by hydrometallurgical processing also plays an important role, especially for simultaneous extraction of other valuable metals like copper and cobalt. Total recoveries of nickel, cobalt, and copper reach more than 99.9% after pressure acid leaching [59].

#### 2.2.3. Pyrometallurgical Processing of Saprolitic Laterite Ore

Several facts related to the processing of saprolitic laterites are noteworthy: (1) the process is usually pyrometallurgical because of high magnesium-silicon content in laterite ore; (2) direct smelting temperatures are higher because nickel is presents in the magnesium silicates; (3) the process is highly energy-intensive as lateritic ores have no significant fuel value to compensate for the heat required; (4) the products are usually ferronickel or NPI as high iron content co-exists in the mineral ore; (5) saprolitic ores have a typical nickel-to-cobalt ratio of 40 to 1, lower than limonitic ores [60,61,62], which is less profitable for extracting Co as a by-product.

Lateritic ores are normally found in tropical climates in deposits below the surface, and hence have a high percentage of free and combined moisture. Therefore, laterite ores need to be dried prior to pyrometallurgical processing. Free moisture is usually removed in a rotary dryer. Calcination at temperatures of 850–1000 °C follows to remove chemically bound moisture and some oxide components. Afterwards, smelting is performed at 1500–1600 °C in an electric arc furnace with the addition of coal or coke as the reducing agent. Typically, all of the nickel oxide and about 60 to 70% of the iron oxide are reduced, and the resulting product is ferronickel. Depending on the smelting conditions, the nickel grades are between 10 to 30% (with average of 15%) with recoveries of 90–95% [63,64].

Recently, studies on processing saprolitic ore have been directed towards low-temperature reduction roasting together with magnetic separation. The pre-roasting process occurs at 650–810 °C. Reduction is accomplished at 1100–1350 °C using reducing agents such as coal, hydrogen, or methane. To enhance the efficiency of selective reduction roasting, the impacts of additives such as chlorides, sulfates, sulfur, alkali oxides, fluorides, sodium carbonate, and sodium hydroxide have been investigated. For 0.8–1.8%-grade saprolitic ores, the nickel concentration is upgraded to average 3.5–7.5% Ni [65,66,67,68,69,70]. This process is attractive in terms of significant energy savings, facile modification for different ores, less demand for water, and environmental friendliness. 

To satisfy the increased demands for class I nickel and cobalt products, alternative processes have been explored to increase Ni grade and Co recovery from saprolitic laterites. Nickel matte obtained from high-temperature smelting is processed via air-blowing to remove sulfur and most iron. The obtained solids are oxidized, followed by water leaching to further remove iron. After the reduction process, nickel (95–97% Ni) is obtained. Alternatively, laterite smelting can be carried out in the presence of sulfur, resulting in sulfide matte. The dried saprolitic ore can also be mixed with concentrated sulfuric acid, followed by roasting and water leaching. The sulfation–roasting–leaching process significantly increases the selective extraction of nickel and cobalt over iron [71,72,73].

#### 2.2.4. Hydrometallurgical Leaching of Limonite or Saprolite Ores

Limonite ores, due to their low nickel grade but very high iron content, are mostly processed via the hydrometallurgical approach. One of the most common hydrometallurgical processes is high-pressure acid leaching (HPAL) based on sulfuric acid. Another is the Caron process, based on ammonia leaching. At present, HPAL is the most prominent industrial approach to extracting nickel due to its effectiveness, short leaching time, low operation cost, high recovery (>90%), and high selectivity. The Caron process consumes more energy compared with HPAL and is only adopted with saprolite ores with high magnesium content.

In a typical HPAL process for laterite, ground laterite particles are mixed with water resulting a slurry with 25–35% solid content. For effective separation of NiO from FeO, strategies of pre-leaching have been investigated which include fine grinding, redox-controlled reactions, addition of salts etc. [74,75,76]. The slurry is then pumped through a series of autoclave compartments containing sulfuric acid. Leaching operates at temperatures 250–270 °C and pressures 3.4 MPa–5.6 MPa. Under these conditions, Ni is extracted in the sulfate solution while Fe, Al, and other impurities precipitate. After several stages of washing, leached nickel is collected, concentrated, and converted into nickel hydroxide precipitate. The obtained solids can be further sulfated, re-leached, separated from remaining impurities through a series of chemical reactions with ammonia, air, and/or sulfide, etc., resulting in high-purity nickel products.

In the Caron process, the finely ground and dried laterite powders are fed into a furnace for reducing combustion at temperatures around 750 °C. Through this process, nickel is reduced to its metallic form while iron remains as oxides and silicates. After being cooled down to temperatures lower than 250 °C in atmosphere, the roasted ores are placed in the ammonia-ammonium carbonate solution. Nickel will form a stable and soluble ammine complex. Although some Fe may also form a soluble iron-ammonia complex, it is confirmed that such an iron-ammonia complex will readily react with oxygen upon exposure to the atmosphere and quickly precipitates as solid iron hydroxide. The nickel solution is selectively collected while the insoluble precipitates containing iron and other minerals are rejected. Through calcination/reduction at intermediate temperatures, ammonia is removed and a solid mixture of nickel carbonate and hydroxide (52% Ni) is produced. After a series of high-temperature reduction/decomposition steps, the products can be high-purity Ni–NiO mixtures or Ni metal.

Atmospheric acid leaching (AAL) and heap leaching (HL) are attractive in terms of their simple process and low capital/energy cost. AAL is an acid leaching process at ambient pressure and temperatures lower than 100 °C. HL is a most traditional mining process, in which very dilute sulfuric acid is slowly applied to crushed nickel ore on a lined pad to dissolve nickel and cobalt. AAL and HL suffer from slow kinetics, slow cycling processes, high consumption of sulfuric acid, and poor selectivity. They are utilized in small-scale, low-grade, or special mines [77,78,79,80].

Innovative technologies, such as pressure acid leach process with additives, hybrid two-stage HPAL-AL, and nitric acid leaching (NAL), have been under investigation [81,82]. Purging oxygen gas during HPAL reduces acid consumption and eliminates unstable precipitates associated with ferrous compounds [83]. Additives like NaCl, Na_2_S_2_O_5_, Na_2_SO_4_, and KCl have been found to accelerate the kinetics of HPAL reactions [84]. HPAL-AL two-stage processes can render mild but selective leaching of Ni and Co from mixed ores. Surfactants like stearyl trimethyl ammonium chloride (STAC) significantly increase the leaching ratios of valuable metals during the two-stage leaching [85,86]. Nitric acid can play a role both as a lixiviant and an oxidant, ensuring high extraction and separation in addition to cost effectiveness and recycling prospects. NAL can be operated at atmospheric or high pressure [87]. Other inorganic acids like HCl and H_3_PO_4_, and organic acids such as citric, lactic, and oxalic acid have also been studied. A mixture of several acids selected from above have shown higher nickel recovery and selectivity [88,89,90,91,92,93]. At present, sulfuric acid leaching is still dominant for the economic considerations.

Bio-leaching has attracted much attention recently, especially for low-grade or complex ores [94]. Unlike in HL, valuable metal compounds in the ore are converted into a water-soluble form with the help of microorganisms and their metabolic products. To increase nickel and cobalt dissolving rates and recovery from nickel-bearing laterites, various key parameters such as direct/indirect bioleaching, types of microorganism species, pH, and the salinity of the culture and growth medium have been studied [95,96,97,98]. Bio-leaching has been successfully applied on a semi-industrial scale. When leaching duration is shortened and metal recovery is further increased, bio-leaching may be more promising for its low environmental effects in addition to low cost in terms of capital, operation, and energy.

## 3. Cobalt

### 3.1. Demands and Land Reserves

Cobalt is mainly used in steels, tools, and catalytic or magnetic materials. Since the 1990s, cobalt has become a key element in superalloys, mainly used in aerospace, marine equipment, and medical appliances. Nowadays, EVs and ESSs have become the largest end use sector in cobalt consumption. In 2022, global consumption of cobalt was 200,000 tonnes, and LIBs accounted for 50% of total cobalt consumed. Implementing the trending RCE scenario, it is projected that demands for cobalt could reach 400,000 tonnes in 2030 [99,100,101]. Cobalt ranks as the 33rd most abundant metal in the earth’s crust, and identified terrestrial cobalt resources are about 25 MTs. However, the global reserves of cobalt in recoverable quantities are only approximately 11.0 MTs and are concentrated in a few countries; for example, the Democratic Republic of Congo (DRC) has 6.0 MTs, followed by Australia (1.7 MTs), Cuba (0.5 MTs), and Indonesia (0.5 MTs).

Unlike nickel, cobalt only presents as a co-existing element within other main metal minerals. Cobalt-bearing land ores include Cu–Co stratiform sediment-hosted (SSH) deposits, Ni–Cu–Co magmatic sulfide deposits, and Ni–Co laterites. Cobalt concentration in these minerals is subjected to chemical and physical changes associated with atmospheric leaching. Cu–Co SSH deposits have a relatively high Co-grade in the range of 0.2–0.9%. Cu–Co SSH deposits can be weathered oxide ore or sulfide ore. Cu–Co oxide ore is mainly carbonates and hydroxides of copper in the form of malachite (Cu(OH)_2_·CuCO_3_). Cobalt is present in the form of heterogenite (CoO(OH)). Cu–Co sulfide ore is mainly chalcopyrite (CuFeS_2_) and covellite (CuS), in which cobalt may co-exist as the sulfide mineral, like carrollite CuCo_2_S_4_ [102,103,104]. In some areas, both exist with the oxide zone extending to a depth of 150 m and a sulfide zone at depths greater than 250 m. In either Ni–Co lateritic or Ni–Cu–Co magmatic ores, Co grade is much lower, varying from 0.01% to 0.2%. Because of the very low Co content in land ores, cobalt is usually a by-product in extracting primary metals, i.e., Cu or Ni.

### 3.2. Cobalt Production from Land Ores

**Figure 5:**
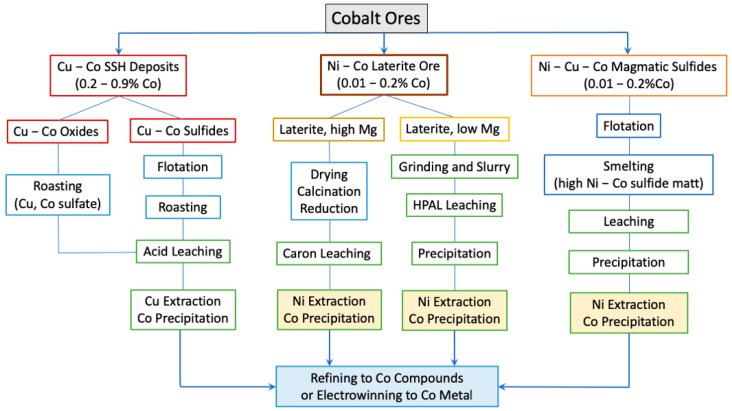
Typical flowchart of different processes of extracting Co from different natural ores.

Global cobalt production is increasing at a pace of 15–20% annually. The DRC, with over 50% of Cu–Co ore reserves, accounts for around 70% of cobalt mining production. Other countries such as Indonesia, with either Ni–Co laterite or Ni–Co–Cu magmatic sulfide ores, contribute 30% of global production. Battery-grade cobalt chemicals are mainly produced from Cu–Co ores. Metallic cobalt can be produced via electrowinning cathodic deposition from Cu–Co sulfide ores, Ni–Co–Cu sulfide ores, and Ni–Co laterites. Figure 5 summarizes the typical flowchart of processing Co from different natural resources.

#### 3.2.1. Processing of Cu−Co SSH Ores

Pre-concentration of Cu–Co sulfide ores can be achieved by flotation. Thiol-type collectors, such as xanthate, dithiophosphate, or dithiocarbamates, are also commonly used with optimum recoveries at pH values of 4–5 [105,106]. Typical flotation recovery rates are about 85% for copper and 75% for cobalt. The conventional flotation process is not directly applicable to oxide and sulfide-oxide mixed ores. Only 75% for copper and 45% for cobalt from oxide ores. For mixed oxide-sulfide ore, recoveries are lower: typical cobalt recovery is about 50–70% from sulfides and 30–40% from oxides [107,108,109]. For oxide ores, the surface of oxide particles is first activated using reagents like sodium sulfide, sodium hydrosulfide, or ammonium sulfide. Sulfur ions adsorbed on oxide particles are much more receptive to sulfhydryl collectors. For sulfide-oxide mixed ores, two-stage flotation is necessary. When carbonaceous minerals are present, special care must be taken to ensure recovery efficiency. A two-step combined sulfide-reverse route has been developed. After sulfide flotation at neutral pH, the second stage is reverse flotation at pH 4.5–5. Overall, Cu–Co recoveries of 93.5% from sulfide and 85.1% from oxide have been achieved [105,110].

After flotation, roasting is carried out when sulfides are oxidized into sulfates. The cobalt and copper sulfates are then subjected to sulfuric acid leaching in the presence of a reducing agent [102]. The reducing agent, like sodium meta-bisulfite (SMBS), will reduce the Co^3+^ ions to more soluble Co^2+^ ions [108]. Cobalt is precipitated as cobalt hydroxide. Further purification may be necessary to get rid of other impurities like iron and aluminum. Electrowinning (EW) can be applied instead of precipitation. This provides a high degree of separation and a high purity of cobalt product. Copper is collected in the solution. After neutralization and a combination of solvent extraction, a number of impurities like Fe, Zn, Mn, etc. are removed [107,111,112].

The ratio of oxide to sulfide in ore is the most important factor in determining the process modification. Two-stage flotation and/or additional sulfuration roasting are the options for mixed ore. When oxide is predominant, the short process route can be leaching–solvent extraction–electrowinning (L/SX/EW). After crushing, the solids are directly fed into a series of atmospheric tanks in the presence of sulfuric acid and a reducing agent like SMBS. A sequence of leaching–solvent extraction–precipitation follows. The leaching process is usually designed to maximize copper recovery with cobalt recovery of less than 75% [113,114].

#### 3.2.2. Processing of Ni–Co Laterite and Ni–Co–Cu Magmatic Sulfide Ores

Ni–Co–Cu magmatic sulfide minerals, similar to Ni sulfide, are processed in the sequence of flotation, smelting, leaching, precipitation, and solution extraction, as described in Section 2.2.3. After the flotation process, nickel and cobalt are separated from copper. Through smelting, concentrated Ni–Co–S matt is obtained, separating from iron slag. It is noted that during smelting, cobalt tends to oxidize more than nickel, resulting in the loss of iron. Adjusting smelting conditions to neutral or reducing atmosphere and the use of slag additives have shown better cobalt recovery [115,116]. The molten Ni–Co sulfide can then be leached using sulfuric acid, ammonia solutions, or hydrochloric acid, which will then be precipitated as sulfate or chloride compounds [117]. In common industrial performance, the recovery rates for cobalt during the flotation and smelting stages average 90% and 50%, respectively.

Cobalt production in conjunction with nickel from Ni–Co laterites has increased significantly in recent years. Although nickel can be extracted from laterite via the pyrometallurgical route, co-extraction of cobalt is not applicable due to the low Co content and loss in the slag. Therefore, the extraction of cobalt from Ni–Co laterites are commonly processed by the hydrometallurgical route as described in Section 2.2.4. The HPAL process is most favorable. Cobalt is recovered by co-precipitation as nickel–cobalt hydroxide or sulfides depending on the details of the leaching processes. Similarly, for laterites with high concentration of Mn, Mg, and Ca, Caron process is selected and cobalt is precipitated as cobalt sulfide. Overall recovery for cobalt is about 93% in HPAL process and less than 80% in Caron process.

Due to the coexistence of nickel and cobalt, which have very similar chemical and physical properties, effective separation of cobalt from nickel is critical and challenging. The most successful process in the mineral industry is based on solution extraction (EX) with the help of organic phosphoric or phosphonic acid [118,119]. The cobalt–X complex is hydrophobic, facilitating its transferability to the organic phase. By contrast, nickel tends to form a nickel–X complex containing one or two water molecules and hence is more hydrophilic. Traditionally, the extractant agent is di(2-ethylhexyl)-phosphoric acid (D2EHPA), whose Co–Ni separation factor is only 10 [120,121]. One recent benchmark reagent is di-2,4,4-trimethylpentylphosphinic acid (DTMPPA, trade name is Cyanex 272) which has a separation factor of ~7000 [122]. When the crude ore is appropriate, solvent extraction can be directly applied to sulfate leaching solutions for separation and purification of nickel and cobalt. This process is more economic because two intermediate precipitation processes are circumvented. There are several plants around the world, like Bulong (Australia) and Goro (New Caledonia) have been operating successfully for the last three decades [123].

## 4. Lithium

### 4.1. Demands and Reserves

Lithium is the most crucial element in electrodes and electrolytes for all Li-based batteries. In 2023, lithium consumption in batteries reached 87% among total global end use. About 4% lithium is used for the production of ceramics and glass (4%), and the remainder is used in the pharmaceutical industry and nuclear reactors, as well as aerospace applications. Dramatic increases in demand have raised serious concerns regarding lithium reserves. It is encouraging to note that worldwide lithium resources (measured and indicated) have increased substantially owing to the continuous exploration and discoveries. In 2024, total lithium reserves reported are about 105 MTs with 90 MTs in brine deposits and 15 Mts in minerals [30].

Lithium brine deposits refer to saline fluids containing high levels of dissolved lithium salt. There are three types of lithium brine deposits: continental, geothermal, and oil field, with lithium contents from 0.01 to 0.2%. Continental saline desert basins (also known as salt lakes, salt flats, or salars) have high lithium concentrations up to 1750 mg/L. The largest Li-rich brine resources with Li concentrations above 1000 mg/L are located in South America, including Bolivia (23 MTs), Argentina (22 MTs), and Chile (11 MTs). Lithium content in geothermal brine and oil/gas field brines are much lower (22–600 mg/L).

Three major lithium minerals, i.e., spodumene, lepidolite, and petalite, are found primarily in pegmatite deposits. Spodumene (4% Li) and petalite (2.3%Li) are made up of Li_2_O, Al_2_O_3_, and SiO_2_, formulated in LiAlSi_2_O_6_ and LiAlSi_4_O_10_, respectively. Lepidolite (about 3%) is a lithium mica mineral with additional K, F, H, etc., formulated as (K(Li,Al)_3_(Al,Si)_4_O_10_(F,OH)_2_). Large deposits of lithium-bearing minerals are concentrated in several geographical regions. Australia has the most spodumene, with estimated reserves of 5.7 MTs, followed by Brazil with 5.4 MTs and Zimbabwe 1.4 million MTs. Brazil and Zimbabwe also have the largest reserves of lepidolite and petalite, respectively. In 2004, a new lithium-containing mineral, named jadarite, was discovered in the Jadar River, Servia. Jadarite is a borosilicate mineral in the form of LiNaSiB_3_O_7_(OH), having a high lithium content (7%). The discovered quantity of the jadarite ore in only a portion of the region is estimated about 136 MTs, with an average content of 1.8% of lithium oxide, which would be the world’s largest lithium solid resources [124,125,126].

### 4.2. Production

**Figure 6:**
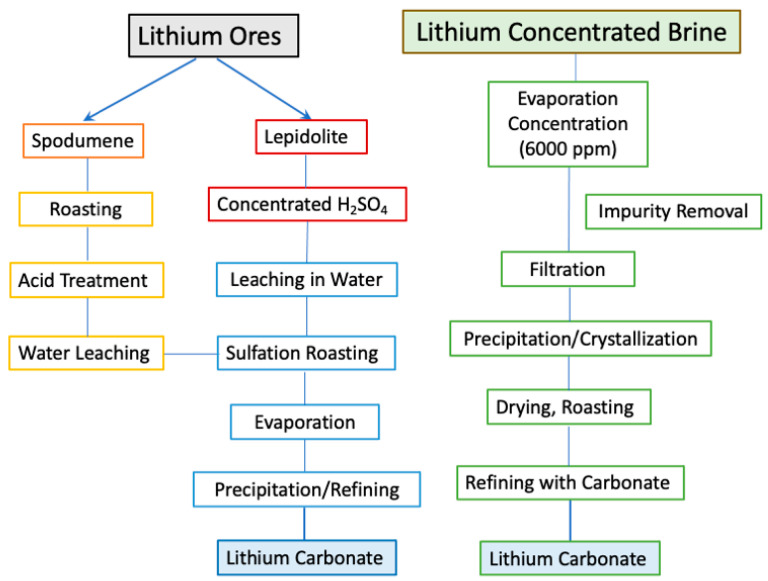
Flowchart of typical different processes of extracting Li from different natural resources (ore and brine).

Lithium is produced from either mineral processing or brine extraction. Figure 6 shows a flowchart of typical extraction of Li from different natural resources. About three quarters of lithium is produced via brine extraction for its larger amount of resources and more economical processing. Lithium must be refined into high-purity battery-grade compounds for synthesizing active cathodes.

#### 4.2.1. Production from Mineral Ores

##### Pre-Concentration

For lithium-bearing mineral ores, heavy medium separation and flotation are the two choices for pre-concentration in plants [127,128]. Heavier spodumene can be separated from lighter gangue silicate minerals (quartz, albite, muscovite, etc.) with the help of heavy media like bromoform. Concentrate grade can be increased to 3.5–6.5% Li_2_O with spodumene recovery of 50–60%. Froth flotation is more favorable for its mature technology, high beneficiation performance, and applicability to complex or low-grade ores. Direct flotation is commonly used for pre-concentrating lithium spodumene from pegmatite ores, in which spodumene is floated and collected in the presence of anionic collectors. Common anionic collectors are oleic acid, sodium oleate, sulfonated, and phosphorated fatty acids. Strong hydrophobicity and adsorption of anionic collectors on the spodumene surface will result in high recovery. Spodumene recoveries in excess of 90% with a concentrate grade of 6.5% Li_2_O was achieved using oleic acid based flotation as early as 1940 [129]. Alternative flotation has been reported using mixed collectors (cationic/anionic, anionic/nonionic) to adding conditioning reagent (multivalent metal ions) [130,131,132].

Relative to spodumene, research on the flotation behavior of lepidolite as well as petalite is scarce but gradually has gradually increased in the past few years [133,134,135,136,137,138,139]. At present, reverse flotation with cationic collectors is common for lepidolite and petalite, in which lithium minerals remain in the slag and are collected. Cationic collectors can be amines, e.g., dodecyl amines. Insights into the flotation mechanism and kinetics are still lacking.

##### Processing Spodumene/Petalite Ores

The standard industrial route to extract lithium from spodumene ore is high-temperature conversion–sulfuric acid digestion–water leaching. Natural spodumene mineral is α-phase (having a monoclinic compact crystal structure), which has low reactivity and hence is very difficult to leach. Therefore, α-spodumene is first converted into β-phase via roasting at 1070–1100 °C. Petalite readily decomposes into β-spodumene and quartz when heated to 1100 °C. Thus, the conventional route for spodumene is also effective for petalite [140,141]. The roasted ore is then mixed with sulfuric acid and treated at 250 °C for about 1 h resulting the formation of Li_2_SO_4_. In this digestion stage, significant excessive sulfuric acid is consumed in side-reactions with impurities such as potassium or sodium present in ore. Water is usually used to leach lithium content, yielding a solution of lithium sulfate. After sulfuration roasting, about 97% of lithium can be recovered [142,143]. This process is the one and only method of extracting lithium from spodumene in the lithium industry. Further pure lithium carbonate with over 99.5% purity can be obtained via evaporation and precipitation.

Direct extraction from α-Spodumene is recently under extensive development to reduce energy consumption. Roasting temperatures can be lowered to 300–500 °C in the presence of alkalis or alkaline salts, such as NaOH, Na_2_CO_3_, Na_2_SO_4_, KOH, Ca(OH)_2_, CaSO_4_, or (NH_4_)_2_SO_4_. The formed LiOH or Li_2_CO_3_ can then be extracted in water leachates. A high recovery in the range of 85–90% has been achieved [144,145,146,147].

##### Production from Lepidolite Ores

Lepidolite has been overshadowed by spodumene for its more complex composition and lower lithium content. Increase in the demand for and price of lithium has stimulated research and production of lithium from the low-grade lithium ore. In lepidolite, a large quantity of impurities is present. On the one hand, F is reactive and can form harmful HF or fluorides during the recovery of Li, which brings complexity in processing. On the other hand, the frequent presence of rare metal ions like Rb^3+^ and Cs^3+^, as well as K^+^ and Al^3+^, can be co-produced, which adds economic benefits for the industry [148,149,150].

Lithium can be extracted from lepidolite directly through acid digestion, and no high temperature roasting is needed. Preconcentrated lepidolite particles are digested using sulfuric acid at elevated temperature to form water-soluble Li_2_SO_4_. Unlike spodumene, the structure of lepidolite is completely destroyed during this process. Consequently, a large quantity of impurities together with lithium are leached simultaneously into the solution. Liu reported that 94.2% Li, 93.7% K, 91.8% Rb, and 89.2% Cs can be leached out [151]. It is reported that HCl is as effective as H_2_SO_4_ in leaching lepidolite ore. Further HCl leaching leads to a relatively simple and safer subsequent purification process [152]. Roasting–water leaching is another optional route to extract Li from Li-bearing mineral clays [150]. Sulfate roasting can be realized at temperatures of 850 °C –1000 °C with the help of Na_2_SO_4_, K_2_SO_4_, CaSO_4_, or FeSO_4_ [150] leading to water-soluble LiKSO_4_ and Li_2_NaK(SO_4_)_2_. Chlorination roasting was also studied for synergistic extraction of Li, Rb, and Cs from lepidolite with the extraction yield of 92.9%, 93.6%, and 93.0%, respectively [153].

#### 4.2.2. Lithium Extraction from Brines

Most continental brines exist under endorheic lake beds in high-altitude regions. Lithium production from these brines is usually achieved via the evaporation–precipitation process [154,155,156,157]. Underground brines are first pumped to the surface and distributed into a series of large shallow open-air ponds. Water is evaporated with the help of natural sunlight and wind until the lithium concentration reaches a level around 6000 ppm, at which point most precipitate in the form of lithium chloride crystals. This traditional process can take a few months. Such brines contain high concentrations of magnesium, potassium, and sodium; therefore, a series of processes is needed to remove these impurities. Lithium chloride (LiCl) can be extracted via precipitation using NaOH and/or roasting with the help of HCl. Lithium chloride can be further roasted and refined into high-purity lithium carbonate (Li_2_CO_3_). Lithium hydroxide (LiOH) is typically produced from refined lithium carbonate. The cost and energy consumption of such production process are about 30–50% less than that of the mined ores. However, this process is time-consuming and has low recovery efficiency. In addition, a large amount of NaOH solution is consumed in precipitation, or HCl is generated during the roasting process. Both have more negative environmental impacts [158]. Salt lake brines usually have a high Mg/Li mass ratio (35:1 up to 1837:1). Neither NaOH or HCl precipitation can effectively remove Mg impurities. Novel reagents, like aluminum-based materials [159], have significantly improved lithium recovery rates (up to 95%) and very high Li/Mg separation performance has been reported.

Research and development on direct extraction technologies are emerging, including a series of precipitation, solvent extraction, sorption, membrane separation, electrochemical pumping, etc. Solvent extraction and ion exchange approaches have exhibited advantages for extracting lithium from high magnesium- and calcium-containing brines. Solvent extraction uses neutral organophosphorus extractants, and tributyl phosphate (TBP)/FeCl_3_ is one promising reagent. When a concentrated HCl solution is used for the subsequent washing and stripping processes, one issue has to be addressed. This reagent readily interacts with HCl, forming HFeCl_4_·2TBP and how to stabilize the reagent and maintain its extraction effectiveness is still under investigation [160,161]. Benefiting from the knowledge gained from LIB development, various lithium metal oxide materials have been adopted to serve as lithium ion sources. Spinel-structured manganese oxides have demonstrated high adsorption rates and superior lithium selectivity, but they usually suffer from dissolution problems [162,163]. Lithium titanium oxide (LTO) has shown low dissolution loss and long recyclability [164]. Such materials have also been applied in electrochemical ion pump systems to capture Li^+^ from brine and to release it into a recovery solution [165,166,167]. Direct lithium extraction has been employed to produce lithium chloride from brines in Argentina and China. Approximately 12% of the world’s lithium supply in 2019 was produced using direct lithium extraction technology. These technologies are more desirable for extracting lithium from brine resources with lower content, e.g., geothermal brine (22–380 mg/L) and oil/gas field brine (50 to 600 mg/L) [168,169,170].

## 5. Ocean Resources and Extraction

In contrast to the very limited land-based reserves, vast amounts of nickel, cobalt, and lithium exist in the ocean. The ocean water system contains an estimated 230 billion tons of lithium. About 290 MTs of nickel and 121 MTs of cobalt have been identified in polymetallic nodules on the abyssal seafloor (4.5 km below the surface) of the ocean. On the slopes and summits of seamounts, ferromanganese crusts are rich in cobalt [171,172]. However, extraction of these valuable metals from the ocean faces both technical and legal challenges. On the one hand, the average lithium concentration in ocean is very low (0.1–0.2 ppm). The traditional evaporation–precipitation approach is not commercially viable due to the extreme low extraction rate. Extracting lithium from oceanic reserves is still in the early stage of research and laboratory experimentation [173,174]. On the other hand, polymetallic nodules are located at greater depths (200 to 6500 m) below water. Seabed resource collecting and lifting will require remotely operated automation systems and advanced hydraulic pumps or bucket systems which are still unavailable [175]. Further, seabed mining could potentially damage sea ecosystems. As of 2024, no permits for commercial deep-sea mining have been granted except a few limited exploration/mining licenses.

## 6. Secondary Resources and Extraction

### 6.1. Recycling Co and Ni from Metal Scraps and Wastes

Secondary resources of Ni and Co include discarded products from magnets or superalloys (over 50% Ni) at their end of life, as well as steel scraps (about 10% Ni), wastes from metallurgical by-products, electronic scraps, and catalysts. Currently, a majority of recycled Ni and Co is from metal scraps and waste superalloys. Methods of recycling metal scraps and wastes can be pyrometallurgy, hydrometallurgy, or pyro-hydrometallurgy [176,177]. Pyrometallurgy of metal scraps encompasses removal of non-metallic substances, re-melting, separation, and purification. Although pyrometallurgy has the advantages of high efficiency and short flow, the recycled products can only serve as additives to steel or other metal alloy production yet are restricted to superalloys. The hydrometallurgical process uses a strong acid solution (such as HCl H_2_SO_4_ or HCl + HNO_3_) to dissolve the valuable metals from scraps/waste into the solution [178,179,180]. Different metallic ions are further separated and purified through a series extraction, precipitation, and ion exchange processes. Purified metals can be revitalized via the hydrometallurgical process. The hydrometallurgical process can become increasingly complex with the uncertain composition of secondary resources, resulting in low recovery efficiency and the generation of effluent and hazardous residues.

### 6.2. Recycling Li, Co, and Ni from Spent LIB Batteries

Although reuse and repurpose can prolong the end life of spent batteries, recycling is the final turning point in the closed-loop flow for material sustainability. Recycling of spent lead acid and nickel–metal hydride (Ni-MH) batteries has been performed for several decades. Nowadays, demand for and the economical values of Li, Co, and Ni have increased significantly. Further legislation regulations have been imposed on LIB manufacturers from spent battery collection and material recovery to recycled content standard. Consequently, academic research and industrial practices regarding recycling LIBs are gaining a great momentum [16,17,18,19,20,21,22,23,24,25,26,180,181,182,183,184,185,186,187,188,189,190,191,192,193].

The universal battery-recycling process comprises the following basic operating steps: (1) physical battery dismantling/sorting; (2) cell deactivation and solvent removal; (3) black mass pyrolysis or whole battery smelting; and (4) hydrometallurgical extraction and separation. Electrode assembly can be separated and sorted from other components of spent batteries. Deactivation and solvent removal can lower electrical and flammable/explosive risks. For LIBs, most liquid electrolytes soaked in electrodes can be evaporated/decomposed at temperatures lower than 300 °C. Pyrolysis at around 700 °C can effectively gasify carbon-based organics and calcine metal-bearing solids. Smelting at higher temperatures (1300–1500 °C) can convert solid materials into lithium-bearing slag and alloys/mattes containing cobalt, nickel, and copper. The hydrometallurgical process can also extract valuable metals (Li, Ni, Co, Cu, Mn, etc.) based on strong acid leaching along with reducing agents (e.g., H_2_SO_4_ and H_2_O_2_). The last refinery step is always to separate individual valuable elements from others based on a series hydrometallurgical processes including precipitation, adsorption, and solvent extraction. Detailed processes need to be modified for specific chemistry and structure of spent batteries [186,187]. Recent research towards advancement in recycling spent LIBs has been reviewed extensively in various aspects. Details can be found in the cited review papers [16,17,18,19,20,21,22,23,24,25,26,180].

Global industrialization in recycling spent LIBs is rapidly advancing, from numerous start-ups emerging to established enterprises expanding. Baum [184] and Latini [185] list current predominant and emerging LIB recycling companies across Asia, Europe, and North America. The core industrial technologies to extract Li, Ni, and Co are categorized as smelting-based pyrometallurgy, leaching-based hydrometallurgy, direct regeneration, and co-precipitation [184,188]. Represented industrial processes, e.g Umicore (Belgium) based on pyrometallurgy and LithoRec (Germany), based on hydrometallurgy, are elaborated in detail in refs. [184,189]. Pyrometallurgy-based processes are currently the most established battery recycling route. They allow for versatile spent batteries to be treated together, regardless of battery chemistry (Ni-MH, Li-ion), electrode composition, and package geometry. One major drawback is the loss of high-value lithium, although it might be collected and refined in downstream recycling operations. Other drawbacks are high energy consumption and net increase in greenhouse gas (GHG) emissions. Hydrometallurgical-based processes, due to their high selectivity, can recover high-purity valuable metal salts of Co, Li, and Ni at low energy costs. However, complex pretreatment is mandatory prior to hydrometallurgical processing, which is a bottleneck in production rate/capacity. Considering environmental impacts, although few toxic gases are emitted, large amounts of wastewater containing acid and hazard chemicals are generated. At present, the recovery efficiencies of Co and Ni from pyrometallurgy are 86 ± 15% and 98 ± 1%, respectively. The recovery efficiencies of Li, Co, and Ni from hydrometallurgy are 95 ± 7%, 95 ± 6%, and 97± 3%, respectively [190]. Compared to pyrometallurgy, hydrometallurgical technology produces high-quality and battery-grade products. 

Industries using direct regeneration or co-precipitation routes are increasing. Ideally, the black mass as-obtained from cathode assembly would be directly regenerated through gentle treatment. If the active cathode materials remain in the morphology and crystal structure, they could be directly re-used for manufacturing new LIBs. This direct regeneration approach may be more suitable for specific cathode chemistry such as LFP and LCO. Alternatively, valuable elements are leached from the black mass and co-precipitated, which can be directly used as precursors to synthesize active cathode. The direct recycling route has great advantages in terms of reducing energy consumption and GHG emission, which has been adopted by several companies. It is noteworthy that direct recycling technologies require more precise classification and disassembly of LIBs. This can add significant labor costs and safety concerns and hinder production capacity. In the near future, advancement in automation and artificial intelligence can be implemented to address these issues. Automated battery recognition, sorting, and disassembly are under research and development [191,192,193].

At present, China and Europe are leading spent LIB recycling. In China, there are six established plants having a total annual capacity of 148,000 tons as of 2020. All are based on hydrometallurgical technology. In Europe, there are 5–6 facilities with an annual capacity over 10,000 tons (as of 2023) distributed across Germany, France, Sweden, Norway, and Belgium. Because half of them are based on pyrometallurgy, the total recovery rate is over 85% for Ni and Co but only 35–42% for Li [190]. Other countries, including the USA, Canada, Japan, and South Korea, also have well-established industries, and many more, like Singapore, Australia, and India have planned for them [183,194,195,196]. However, recycling LIBs in developing countries is still in its infancy due to the lack of regulatory frameworks and financial investments. These hinder the recycling plant capacities and/or management infrastructures. The efficiency and profit depend on the price of raw material, recycling process, and battery collection rate. Feasible infrastructure is needed to ensure safety and environmentally friendly production. It is estimated that the capital cost of a 100 tonne per annum capacity for pyrometallurgical, for hydrometallurgical, black mass processes are 1.06, 0.66, and 0.18 million US dollars [197]. For a 9000 tonne per year capacity, the total capital cost is 3.5 million USD (only 21.6% of the low capacity). Current recycling facilities in Latin America and the Caribbean are mostly for mechanical treatment (shredding) and the following separation of different fractions (black mass, copper, and aluminum) [198]. The costs of initial investment and recycling production are still high, but they are expected to decrease significantly when higher recycling capacities are installed.

## 7. Conclusions

Broad implementation of renewable clean energy technologies with the aim of reducing carbon emission and pollution has dramatically increased the demands for rechargeable energy conversion and storage systems. Lithium-ion batteries are presently dominant in applications to electric vehicles and battery energy storage systems. Exponential increase in demand for LIBs have raised concerns and pressures on resources and production on the key elements such as Li, Ni, and Co. According to the latest data, the identified economical land ores for Li, Ni, and Co are 105 MTs, 130 MTs, and 25 MTs, respectively, and the present annual consumptions of them are 0.18 MTs, 0.26 MTs, and 3.4 MTs, respectively. Based on zero-emission scenarios, demand will increase by approximately 100% for lithium and 50% for Co and Ni in 2030. Present resources will be sustainable for several decades. Meanwhile, exploration activities and recycling from waste are expanding. Although still in their infancy, advanced batteries based on alternative chemistry, such as Li-sulfur and sodium-ion batteries, can offer alternative candidates which can alleviate the demands and resource depletion.

Technologies from mining to extraction and recycling of these valuable metals are crucial to advancing towards improving recovery efficiency and reducing energy consumption but meanwhile to minimize environmental damage. Most existing pyrometallurgical processes consume significant amount of energy and emit hazardous gases. Hydrometallurgical processes utilize large amounts of alkaline or acidic media in combination with reducing agents, generating hazardous waste stream. The environmental impact will be local air/water/land pollution and as a consequence in the long term will lead to drought, deforestation, reduced agricultural yield, fatal ailments, etc. [199,200,201,202,203,204,205]. From this point of view, it is urgent to develop more energy-effective and environmentally friendly processes in line with the green world concept to minimize net global carbon emission.

## Figures and Tables

**Table 1:** Comparison of traditional and advanced pre-concentration approaches.

Methods	Property and Facility	Example Results
Tradition [34]	Size, screening	0.54% to 1.1% at 62% recovery [35]
Gravity [36]	Density, centrifugation or dense media	1.1% Ni to 1.5% with 70% recovery using Falcon [37] 0.43% to 0.66% with 87% recovery using DMS [38]
Magnetic [39]	Magnetism, magnetic field w/o roasting oven	0.89% to 1.08% with 57% recovery [34] laterite ore Ni 0.72%, Co 0.029%, Fe 8.65%; ferronickel 16.16% Ni, 73.67% Fe with 90% Ni recovery [40]

## Data Availability

No new data were created or analyzed in this study. Data sharing is not applicable to this article.

## References

[1] International Energy Agency (2023). Global EV Outlook 2023: Catching up with Climate Ambitions.

[2] International Energy Agency (2024). Global EV Outlook 2024: Moving towards Increased Affordability.

[3] Zhou Y., Gohlke D., Rush L., Kelly J., Dai Q. (2021). Lithium-Ion Battery Supply Chain for e-Drive Vehicles in the United States: 2010–2020.

[4] Cohen A. Manufacturers Are Struggling to Supply Electric Vehicles with Batteries. https://www.forbes.com/sites/arielcohen/2020/03/25/manufacturers-are-struggling-to-supply-electric-vehicles-with-batteries/.

[5] Grand View Research (2022). Market Analysis Report: Energy Storage Systems Market Size, Share & Trends Analysis Report by Technology (Pumped Storage, Electrochemical Storage, Electromechanical Storage, Thermal Storage), by Region, and Segment Forecasts, 2023–2030.

[6] Mckinsey and Company (2023). Market Analysis Report: Enabling Renewable Energy with Battery Energy Storage Systems.

[7] Linden D., Reddy T.B., Thomas B. (2001). Handbook of Batteries.

[8] Ralls A.M., Leong K., Clayton J., Fuelling P., Mercer C., Navarro V., Menezes P.L. (2023). The Role of Lithium-ion batteries in the growing trend of electric vehicles. Materials.

[9] Zhao Y., Pohl O., Bhatt A.I., Collis G.E., Mahon P.J., Rüther T., Hollenkamp A.F. (2021). A Review on Battery Market Trends, Second-Life Reuse, and Recycling. Sustain. Chem..

[10] Zhou W., Cleaver C., Dunant C., Allwood J. (2023). Cost, range anxiety and future electricity supply: A review of how today’s technology trends may influence the future uptake of BEVs. Renew. Sustain. Energy Rev..

[11] Ragozzino A. (2022). What’s Next for Electric-Vehicle Battery Technology?.

[12] Biswal B.K., Balasubramanian R. (2023). Recovery of valuable metals from spent lithium-ion batteries using microbial agents for bioleaching: A review. Front. Microbiol..

[13] Saaid F.I., Kasim M.F., Winie T., Elong K.A., Azahidi A., Basri N.D., Yaakob M.K., Mastuli M.S., Shaffee S.N.A., Zolkiffly M.Z. (2024). Ni-rich lithium nickel manganese cobalt oxide cathode materials: A review on the synthesis methods and their electrochemical performances. Heliyon.

[14] Winslow K.M., Laux S.J., Townsend T.G. (2018). A review on the growing concern and potential management strategies of waste lithium-ion batteries. Resour. Conserv. Recycl..

[15] Xu C., Dai Q., Gaines L., Hu M., Tukker A., Steubing B. (2020). Future materials demand for automotive lithium-based batteries. Commun. Mater..

[16] Bernardes A.M., Espinosa D.C.R., Tenório J.A.S. (2004). Recycling of batteries: A review of current processes and technologies. J. Power Sources.

[17] Li L., Zhang X., Li M., Chen R., Wu F., Amine K., Lu J. (2018). The recycling of spent lithium-ion batteries: A review of current processes and technologies. Electrochem. Energy Rev..

[18] Lv W., Wang Z., Cao H., Sun Y., Zhang Y., Sun Z. (2018). A Critical Review and Analysis on the Recycling of Spent Lithium-Ion Batteries. ACS Sustain. Chem. Eng..

[19] Jena K.K., AlFantazi A., Mayyas A.T. (2021). Comprehensive Review on Concept and Recycling Evolution of Lithium-Ion Batteries (LIBs). Energy Fuels.

[20] Zhang J., Azimi G. (2022). Recycling of lithium, cobalt, nickel, and manganese from end-of-life lithium-ion battery of an electric vehicle using supercritical carbon dioxide. Resour. Conserv. Recycl..

[21] Dobó Z., Dinh T., Kulcsár T. (2023). A review on recycling of spent lithium-ion batteries. Energy Rep..

[22] Pan C., Shen Y. (2023). Pyrometallurgical recycling of spent lithium-ion batteries from conventional roasting to synergistic pyrolysis with organic wastes. J. Energy Chem..

[23] Ren Z., Li H., Yan W., Lv W., Zhang G., Lv L., Sun L., Gao W. (2023). Comprehensive evaluation on production and recycling of lithium-ion batteries: A critical review. Renew. Sustain. Energy Rev..

[24] Li P., Luo S., Zhang L., Liu Q., Wang Y., Lin Y., Xu C., Guo J., Chealic P., Xia X. (2024). Progress, challenges, and prospects of spent lithium-ion batteries recycling: A review. J. Energy Chem..

[25] Zhao Q., Sun K., Wang X., Wang Q., Wang J. (2024). Examining green-sustainable approaches for recycling of lithium-ion batteries. DeCarbon.

[26] Biswal B.K., Zhang B., Tran P., Zhang J., Balasubramanian R. (2024). Recycling of spent lithium-ion batteries for a sustainable future: Recent advancements. Chem. Soc. Rev..

[27] British Geological Survey (2008). Nickel. https://core.ac.uk/download/pdf/58868.pdf.

[28] Schmidt T., Buchert M., Schebek L. (2016). Investigation of the primary production routes of nickel and cobalt products used for Li-ion batteries. Resour. Conserv. Recycl..

[29] Meshram P., Pandey B.D. (2019). Advanced review on extraction of Nickel from primary and secondary sources by pre-treatment, leaching, and separation: A comprehensive review. Metall. Rev..

[30] U.S. Geological Survey (2024). Mineral Commodity Summaries.

[31] Zhao K., Gao F., Yang Q. (2022). Comprehensive review on metallurgical upgradation processes of nickel sulfide ores. J. Sustain. Metall..

[32] Stankovic S., Topic S.S., Okic M., Markovic B., Friedrich B. (2020). Review of the past, present, and future of the hydrometallurgical production of nickel and cobalt from lateritic ores. Metall. Mater. Eng..

[33] (2019). Munali Nickel Mine’s “Game-Changing” Dense Media Separator (DMS) Is Flying the Flag High for Zambia. https://miningforzambia.com/munali-nickel-mines-game-changing-dense-media-separator-dms-is-flying-the-flag-high-for-zambia/.

[34] Quast K., Connor J.N., Skinner W., Robinson D.J., Addai-Mensh J. (2015). Preconcentration strategies in the processing of nickel laterite ores Part 1: Literature review. Miner. Eng..

[35] Rao K.A., Sreenivas T., Natarajan R., Rao N.K. (1995). Preconcentration of nickel values from lateritic chromite ore overburden, Sukinda, Orissa, India. Miner. Process. Extract. Metall. Rev..

[36] Nayak A., Jena M.S., Mandre N.R. (2021). Application of Enhanced Gravity Separators for Fine Particle Processing: An Overview. J. Sustain. Metall..

[37] Arrokhpay S., Filippov L., Fornasiero D. (2019). Pre-concentration of nickel in laterite ores using physical separation methods. Min. Eng..

[38] Pillay K., Mainza A.N., Chetty D., Becker M. (2022). Mineralogical Factors Affecting the Dense Medium Separation of Nickel Sulfide Ores. Minerals.

[39] Iranmanesh M., Hulliger J. (2017). Magnetic separation: Its application in mining, waste purification, medicine, biochemistry and chemistry. Chem. Soc. Rev..

[40] Xiao J., Ding W., Peng Y., Chen T., Zou K., Wang Z. (2020). Extraction of Nickel from Garnierite Laterite Ore Using Roasting and Magnetic Separation with Calcium Chloride and Iron Concentrate. Minerals.

[41] Srdjan Bulatovic M. (2007). Handbook of Flotation Reagents Chemistry, Theory and Practice: Flotation of Sulfide Ores.

[42] Ian D., Wilson Colin F. (2000). Poole Michael Cooke. Encyclopedia of Separation Science.

[43] Lotter N.O., Bradshaw D.J. (2010). The formulation and use of mixed collectors in sulphide flotation. Miner. Eng..

[44] Eliana Mano S., Caner L., Petit S., Arthur Chaves P., André Mexias S. (2019). Ni-smectitic ore behaviour during the Caron process. Hydrometallurgy.

[45] Pérez-Garibay R., Ramírez-Aguilera N., Bouchard J., Rubio J. (2014). Froth flotation of sphalerite: Collector concentration, gas dispersion and particle size effects. Miner. Eng..

[46] Masiya T.T., Nheta W. Flotation of Nickel-Copper Sulphide Ore: Optimisation of Process Parameters Using Taguchi Method. Proceedings of the International Conference on Mining, Material and Metallurgical Engineering.

[47] Chernousenko V.E., Neradovsky Y.N., Kameneva Y.S., Vishnyakova I.N., Mitrofanova G.V. (2018). Increasing Efficiency of Pechenga Rebellious, Copper–Nickel Sulphide Ore Flotation. J. Min. Sci..

[48] Long T., Zhao H., Wang Y., Yang W., Deng S., Xiao W., Lan X., Wang Q. (2022). Synergistic mechanism of acidified water glass and carboxymethyl cellulose in flotation of nickel sulfide ore. Miner. Eng..

[49] Faris N., Pownceby M.I., Bruckard W.J., Chen M. (2023). The Direct Leaching of Nickel Sulfide Flotation Concentrates—A Historic and State-of-the-Art Review Part I: Piloted Processes and Commercial Operations. Miner. Process. Extr. Metall. Rev..

[50] Anzoom S.J., Bournival G., Ata S. (2024). Coarse particle flotation: A review. Miner. Eng..

[51] Mweene L., Gomex-Flores A., Jeong H.E., Ilyas S., Kim H. (2023). Challenges and Future in Ni Laterite Ore Enrichment: A Critical Review. Miner. Process. Extr. Metall. Rev..

[52] Farrokhpay S., Filippov L. (2016). Challenges in processing nickel laterite ores by flotation. Int. J. Miner. Process..

[53] Farrokhpay S., Fornasiero D., Filippov L. (2018). Upgrading nickel in laterite ores by flotation. Miner. Eng..

[54] Quast K., Connor J.N., Skinner W., Robinson D.J., Addai-Mensh J. (2015). Preconcentration strategies in the processing of nickel laterite ores part 3: Flotation testing. Miner. Eng..

[55] Zappala L., McDonald R., Powceby M.I. (2023). Nickel Laterite Beneficication and Potential for Upgrading Using High Temperature Methods: A Review. Miner. Process. Extr. Metall. Rev..

[56] Fang X., Peng z., Yin T., Rao M., Li G. (2024). Microwave Treatment of Copper–Nickel Sulfide Ore for Promotion of Grinding and Flotation. Metals.

[57] Mu W., Cui F., Huang Z., Zhai Y., Xu Q., Luo S. (2018). Synchronous extraction of nickel and copper from a mixed oxide-sulfide nickel ore in a low-temperature roasting system. J. Clean. Prod..

[58] Cui F., Mu W., Wang S., Xin H., Xu Q., Zhai Y., Luo S. (2018). Sodium sulfate activation mechanism on co-sulfating roasting to nickel copper sulfide concentrate in metal extractions, microtopography and kinetics. Min. Eng..

[59] Huang K., Li Q.W., Chen J. (2007). Recovery of copper, nickel and cobalt from acidic pressure leaching solutions of low-grade sulfide flotation concentrates. Min. Eng..

[60] Marzoughi O., Pickles C.A. (2024). Solid state reduction and magnetic separation of nickeliferous laterite ores: Review and analysis. J. Ind. Eng. Chem..

[61] Keskinkilic E. (2019). Nickel laterite smelting processes and some examples of recent possible modifications to the conventional route. Metals.

[62] Nurjaman F., Astuti W., Bahfie F., Suharno B. (2021). Study of selective reduction in lateritic nickel ore: Saprolite versus limonite. Mater. Today Proc..

[63] Norgate T., Jahanshahi S. (2010). Low grade ores—Smelt, leach or concentrate?. Miner. Eng..

[64] Vahed A., Mackey P.J., Warner E.M.A. A Review of Nickel Pyrometallurgy Over the Past 50 Years with Special Reference to the Former Inco Ltd and Falconbridge Ltd. Proceedings of the 5th International Symposium on Nickel and Cobalt.

[65] Ma B., Xing P., Yang W., Wang C., Chen Y., Wang H. (2017). Solid-state metalized reduction of magnesium-rich low-nickel oxide ores using coal as the reductant based on thermodynamic analysis. Metall. Mater. Trans. B.

[66] Lv X., Lv W., Liu M., You Z., Lv X., Bai C. (2018). Effect of sodium sulfate on preparation of ferronickel from nickel laterite by carbothermal reduction. ISIJ Int..

[67] Hang G., Xue Z., Wang J., Wu Y. (2020). Mechanism of calcium sulphate on the aggregation and growth of ferronickel particles in the self-reduction of saprolitic nickel laterite ore. Metals.

[68] Liu S., Yang C., Yang S., Yu Z., Wang Z., Yan K., Li J., Liu X. (2021). A robust recovery of Ni from laterite ore promoted by sodium thiosulfate through hydrogen-thermal reduction. Front. Chem..

[69] Zulhan Z., Shalat W. (2021). Evolution of ferronickel particles during the reduction of low-grade saprolitic laterite nickel ore by coal in the temperature range of 900–1250 °C with the addition of CaO-CaF_2_-H_3_BO_3_. Int. J. Miner. Metall. Mater..

[70] Yang W., Ma B., Li X., Hu D., Wang C., Wang H. (2022). Transferring behavior and reaction kinetics of saprolitic laterite during metalized reduction in the presence of calcium fluoride. Miner. Eng..

[71] Guo X., Li D., Park K., Tian Q., Wu Z. (2009). Leaching behavior of metals from a limonitic nickel laterite using a sulfation–roasting–leaching process. Hydrometallurgy.

[72] Basturkcu H., Acarkan N. (2016). Saparation of Nickel and Iron from Laterite ore using digestion-roating-leaching-precipitation Process. Physicochem. Probl. Miner. Process..

[73] Ribeiro P.P.M., Santos I.D.D., Neumann R., Fernades A., Dutra A.J.B. (2021). Roasting and Leaching Behavior of Nickel Laterite Ore. Metall. Mater. Trans. B.

[74] Whttington B.I., Muir D. (2000). Pressure acid leaching of nickel laterites: A Review, Mineral Processing and Extractive Metallurgy Review. Int. J..

[75] Akbar Rhamdhani M., Chen J., Hidayat T., Jak E., Hayes P. (2009). Advances in research on nickel production through the Caron process. Proc. EMC.

[76] Gultom T., Sianipar A. (2020). High pressure acid leaching: A newly introduced technology in Indonesia. Environ. Earth Sci..

[77] McDonald R.G., Whittington B.I. (2008). Atmospheric acid leaching of nickel laterites review: Part I. Sulphuric acid technologies. Hydrometallurgy.

[78] Luo J., Li G., Rao M., Peng Z., Zhang Y., Jiang T. (2015). Atmospheric leaching characteristics of nickel and iron in limonitic laterite with sulfuric acid in the presence of sodium sulfite. Miner. Eng..

[79] Oxley A., Smith M.E., Caceres O. (2016). Why heap leach nickel laterites?. Miner. Eng..

[80] Agatzini-Leonardou S., Oustadakis P., Dimaki D., Zafiratos J., Tsakiridis P., Karidakis T., Frogoudakis E., Drougas J. (2021). Heap leaching of greek low-grade nickel oxide ores by dilute sulphuric acid at a pilot-plant scale. Mater. Proc..

[81] Robinson D.J., McDonald R., Zhang W., McCarthy F. Developments in the hydrometallurgical processing of laterites. Proceedings of the COM 2017-Nickel/Cobalt Hydrometallurgy Symposium.

[82] Pandey N., Tripathy S.K., Patra S.K., Jha G. (2023). Recent Progress in Hydrometallurgical Processing of Nickel Lateritic Ore. Trans. Indian Inst. Met..

[83] Johnson J.A., Cashmore B.C., Hockridge R.J. (2005). Optimization of nickel extraction from laterite ores by high pressure acid leaching with addition of sodium sulphate. Miner. Eng..

[84] Loveday B.K. (2008). The use of oxygen in high pressure acid leaching of nickel laterites. Miner. Eng..

[85] Zhang P., Sun L., Wang H., Cui J., Hao J. (2019). Surfactant-assistant atmospheric acid leaching of laterite ore for the improvement of leaching efficiency of nickel and cobalt. J. Clean. Prod..

[86] Zhang P., Wang H., Hao J., Cui J. (2021). Reinforcement of the two-stage leaching of laterite ores using surfactants. Front. Chem. Sci. Eng..

[87] He F., Ma B., Qi Z., Wang C., Chen Y., Hu X. (2023). Enhanced extraction of nickel from limonitic laterite via improved nitric acid pressure leaching process. Miner. Eng..

[88] Lakshmanan V.I., Sridhar R., DeLaat R., Chen J., Halim M.A., Roy R. (2013). Extraction of nickel, cobalt and iron from laterite ores by mixed chloride leach process. InNi–Co.

[89] Top S., Kusunoglu S., Ichlas Z.T. (2020). Effects of leaching parameters on the dissolution of nickel, cobalt, manganese and iron from Caldag lateritic nickel ore in hydrochloric acid solution. Can. J. Metall. Mater. Sci..

[90] Hosseini Nasab M., Noaparast M., Abdollahi H. (2020). Dissolution of Nickel and Cobalt from Iron-Rich Laterite Ores Using Different Organic Acids. J. Min. Environ. (JME).

[91] Li G., Zhou Q., Zhu Z., Luo J., Rao M., Peng Z., Jiang T. (2018). Selective leaching of nickel and cobalt from limonitic laterite using phosphoric acid: An alternative for value-added processing of laterite. J. Clean. Prod..

[92] Astuti W., Hirajima T., Sasaki K., Okibe N. (2015). Comparison of effectiveness of citric acid and other acids in leaching of low-grade Indonesian saprolitic ores. Miner. Eng..

[93] Astuti W., Nurjaman F., Mufakhir F.R., Sumardi S., Avista D., Wanta K.C., Petrus H.T.B.M. (2023). A novel method: Nickel and cobalt extraction from citric acid leaching solution of nickel laterite ores using oxalate precipitation. Miner. Eng..

[94] Abdollahi H., Nasab M.H., Yadollahi A. (2024). Bioleaching of Lateritic Nickel Ores. Biotechnological Innovations in the Mineral-Metal Industry.

[95] Le L., Tang J., Ryan D., Valix M. (2006). Bioleaching nickel laterite ores using multi-metal tolerant Aspergillus foetidus organism. Miner. Eng..

[96] Yang Y., Ferrier J., Csetenyi L., Gadd G.M. (2019). Direct and indirect bioleaching of cobalt from low grade laterite and pyritic ores by Aspergillus niger. Geomicrobiol. J..

[97] Nasab M.H., Noaparast M., Abdollahi H., Ali Amoozegar M. (2020). Indirect bioleaching of Co and Ni from iron rich laterite ore, using metabolic carboxylic acids generated by *P. putida*, *P. koreensis*, *P. bilaji* and *A. niger*. Hydrometallurgy.

[98] Carpen H.L., Giese E.C. (2022). Enhancement of nickel laterite ore bioleaching by Burkholderia sp. using a factorial design. Appl. Water Sci..

[99] U.S. Geological Survey (2023). Mineral Commodity Summaries: Cobalt.

[100] Fu X., Beatty D.N., Gaustad G.G., Ceder G., Roth R., Kirchain R.E., Bustamante M., Babbitt C., Olivetti E.A. (2020). Perspectives on Cobalt Supply through 2030 in the Face of Changing Demand. Environ. Sci. Technol..

[101] Savinova E., Evans C., Lèbre É., Stringer M., Azadi M., Valenta R.K. (2023). Will global cobalt supply meet demand? The geological, mineral processing, production and geographic risk profile of cobalt. Resour. Conserv. Recycl..

[102] Dehaine Q., Tijsseling L.T., Glass G.K., Tormane T. (2021). Geometallurgy of cobalt ores: A review. Miner. Eng..

[103] Picazo-Rodriguez N., Toro N., Roman M., Soriano D., Madrid F., Jamett J., Galvez E., Cedillos J. (2023). Cobalt metal: Overview of deposits, reserves, processings, and recycling. Preprints.

[104] Huang Y., Chen P., Shu X., Fu B., Peng W., Liu J., Cao Y., Zhu X. (2024). Extraction and recycling technologies of cobalt from primary and secondary resources. Int. J. Miner. Metall. Mater..

[105] Tijsseling L.T., Dehaine Q., Rollinson G.K., Glass H.J. (2019). Flotation of mixed oxide sulphide copper-cobalt minerals using xanthate, dithiophosphate, thiocarbamate and blended collectors. Miner. Eng..

[106] Tijsseling L.T., Dehaine Q., Rollinson G.K., Glass H.J. (2020). Mineralogical Prediction of Flotation Performance for a Sediment-Hosted Copper–Cobalt Sulphide Ore. Minerals.

[107] Fisher K.G., Treadgold L.G. Design Considerations for the Cobalt Recovery Circuit of the KOL (KOV) Copper/Cobalt Refinery, DRC. Proceedings of the ALTA Nickel-Cobalt Conference.

[108] Crundwell F.K., Moats M.S., Ramachandran V., Robinson T.G., Davenport W.G., Crundwell F.K., Moats M.S., Ramachandran V., Robinson T.G., Davenport W.G. (2011). Production of Cobalt from the Copper–Cobalt Ores of the Central African Copperbelt. Extractive Metallurgy of Nickel, Cobalt and Platinum Group Metals.

[109] Ou L.M., Yin B.Y. (2011). A Flotation Technique for a Sulfide-Oxidized Cu-Co Mixed Ore. Adv. Mater. Res..

[110] Dehaine Q., Filippov L.O., Filippova I.V., Tijsseling L.T., Glass H.J. (2021). Novel approach for processing complex carbonate-rich copper-cobalt mixed ores via reverse flotation. Miner. Eng..

[111] Swartz B., Donegan S., Amos S.R. (2009). Processing considerations for cobalt recovery from Congolese copperbelt ores. Hydrometallurgy Conference.

[112] Oraby E., Deng Z., Li H., Eksteen J. (2023). Selective extraction of nickel and cobalt from disseminated sulfide flotation cleaner tailings using alkaline glycine-ammonia leaching solutions. Miner. Eng..

[113] Shengo M.L., Kime M.-B., Mambwe M.P., Nyembo T.K. (2019). A review of the beneficiation of copper-cobalt-bearing minerals in the Democratic Republic of Congo. J. Sustain. Min..

[114] Stuurman S., Ndlovu S., Sibanda V. (2014). Comparing the extent of the dissolution of copper-cobalt ores from the DRC Region. Inst. Min. Metall..

[115] Morcali M.H., Khajavi L.T., Dreisinger D.B. (2017). Extraction of nickel and cobalt from nickeliferous limonitic laterite ore using borax containing slags. Int. J. Miner. Process..

[116] Dong J., Wei Y., Zhou S., Li B., Yang Y., Mclean A. (2018). The Effect of Additives on Extraction of Ni, Fe and Cofrom Nickel Laterite Ores. JOM.

[117] Peek E., Åkre T., Asselin E. (2009). Technical and business considerations of cobalt hydrometallurgy. Sustain. Process..

[118] Preston J.S. (1982). Solvent extraction of cobalt and nickel by organophosphorus acids I. Comparison of phosphoric, phosphonic and phosphonic acid systems. Hydrometallurgy.

[119] Sole K.C. (2018). The Evolution of Cobalt–Nickel Separation and Purification Technologies: Fifty Years of Solvent Extraction and Ion Exchange. Extraction 2018 Conference.

[120] Flett D.S. (2004). Cobalt-Nickel separation in hydrometallurgy: A review. Chem. Sustain. Dev..

[121] Flett D.S. (2005). Solvent extraction in hydrometallurgy: The role of organophosphorus extractants. J. Organomet. Chem..

[122] Gupta B., Mudhar N., Singh I. (2007). Separations and recovery of indium and gallium using bis(2,4,4-trimethylpentyl)phosphinic acid (Cyanex 272). Sep. Purif. Technol..

[123] Kurunoglu S., Kaya M. (2019). Hydrometallurgical processing of Nickel laterites—A brief overview on the use of solvent extraction and Nickel/Cobalt Project for the separation and purification of nickle and cobalt. Mining.

[124] Meshram P., Pandey B.D., Mankhand T.R. (2014). Extraction of lithium from primary and secondary sources by pre-treatment, leaching, and separation: A comprehensive review. Hydrometallurgy.

[125] Mohr S.H., Mudd G.M., Giurco D. (2012). Lithium resources and production: Critical assessment and global projections. Minerals.

[126] Tadesse B., Makuei F., Albijanic B., Dyer L. (2019). The beneficiation of lithium minerals from hard rock ores: A review. Miner. Eng..

[127] Kundu T., Rath S.S., Kanta Das S., Parhi P.K., Angadi S.I. (2023). Recovery of lithium from spodumene-bearing pegmatites: A comprehensive review on geological reserves, beneficiation, and extraction. Powder Technol..

[128] Norman J., Gieseke E.W. (1940). Beneficiation of Spodumene Rock by froth flotation. Trans. Am. Inst. Min. Metall. Eng. Min. Pract..

[129] Xu L., Wu H., Dong F., Wang L., Wang Z., Xiao J. (2013). Flotation and adsorption of mixed cationic/anionic collectors on muscovite mica. Miner. Eng..

[130] Wang Y., Zhu G., Yu F., Lu D., Wang L., Zhao Y., Zheng H. (2018). Improving spodumene flotation using a mixed cationic and anionic collector. Physicochem. Prob. Miner. Process..

[131] Yu F., Wang Y., Zhang L., Zhu G. (2015). Role of oleic acid-molecular complexes in the flotation of spodumene. Miner. Eng..

[132] Liu W., Zhang S., Wang W., Zhang J., Yan W., Deng J., Huang Y. (2015). The effects of Ca(II) and Mg(II) ions on the flotation of spodumene using NaOL. Miner. Eng..

[133] Filippov L.O., Filippova I.V., Crumiere G., Sousa R., Leite M.M., de Sousa A.B., Korbel C., Tripathy S.K. (2022). Separation of lepidolite from hard-rock pegmatite ore via dry processing and flotation. Miner. Eng..

[134] Korbel C., Filippova I.V., Filippov L.O. (2023). Froth flotation of lithium micas—A review. Miner. Eng..

[135] Liu K. (2023). Research progress in flotation collectors for lepodolite monierals: An overview. Miner. Process. Extr. Metall. Rev..

[136] Vieceli N., Durão F.O., Guimarães C., Nogueira C.A., Pereira M.F.C., Margarido F. (2016). Kinetic approach to the study of froth flotation applied to a lepidolite ore. Int. J. Miner. Metall. Mater..

[137] Huang Z., Shuai S., Wang H., Liu R., Zhang S., Chen C., Hu Y., Yu X., He G., Fu W. (2022). Froth flotation separation of lepidolite ore using a new Gemini surfactant as the flotation collector. Sep. Purif. Technol..

[138] Li J., Nie G., Li J., Zhu Z., Wang Z. (2022). Flotation separation of quartz and dolomite from collophane using sodium N-dodecyl-β-amino propionate and its adsorption mechanism. Colloids Surf. A.

[139] Zhou J., Chen Y., Li W., Song Y., Xu W., Li K., Zhang Y. (2023). Mechanism of Modified Ether Amine Agents in Petalite and Quartz Flotation Systems under Weak Alkaline Conditions. Minerals.

[140] Liu Y., Ma B., Lv Y., Wang C., Chen Y. (2023). A review of lithium extraction from natural resources. Int. J. Miner. Metall. Mater..

[141] Sitando O., Crouse P.L. (2012). Processing of a Zimbabwean petalite to obtain lithium carbonate. Int. J. Miner. Process..

[142] Rioyo J., Tuset S., Grau R. (2020). Lithium Extraction from spodumene by traditional sulfuric acid process: A review. Miner. Process. Extr. Metall. Rev..

[143] Dessemond C., Lajoie-Leroux F., Soucy G., Laroche N., Magnan J.-F. (2019). Spodumene: The Lithium Market. Resources and Processe. Minerals.

[144] Karrecha A., Azadia M.R., Elchalakania M., Shahinb M.A., Seibic A.C. (2020). A review on methods for liberating lithium from pegmatities. Miner. Eng..

[145] Song Y., Zhao T., He L., Zhao Z., Liu X. (2019). A promising approach for directly extracting lithium from α-spodumene by alkaline digestion and precipitation as phosphate. Hydrometallurgy.

[146] Han S., Sagzhanov D., Pan J., Hassas B.V., Rezaee M., Akbari H., Mensah-Biney R. (2022). Direct Extraction of Lithium from α-Spodumene by Salt Roasting−Leaching Process. ACS Sustain. Chem. Eng..

[147] Maliachova K., Doukas N., Tsakiri D., Samouhos M., Sakellariou L., Douni I., Taxiarchou M., Paspaliaris I. (2023). Li Extraction from a-Spodumene Concentrate via Carbonizing Calcination. Mater. Proc..

[148] Gao T., Fan N., Chen W., Dai T. (2023). Lithium extraction from hard rock lithium ores (spodumene, lepidolite, zinnwaldite, petalite): Technology, resources, environment and cost. China Geol..

[149] Li H., Eksteen J., Kuang G. (2019). Recovery of lithium from mineral resources: State-of-the-art and perspectives—A review. Hydrometallurgy.

[150] Zhao H., Wang Y., Cheng H. (2023). Recent advances in lithium extraction from lithium-bearing clay minerals. Hydrometallurgy.

[151] Liu J.-L., Yin Z.-L., Li X.-H., Hu Q.-Y., Liu W. (2019). Recovery of valuable metals from lepidolite by atmosphere leaching and kinetics on dissolution of lithium. Trans. Nonferrous Met. Soc. China.

[152] Liu J.L., Yin Z.L., Liu W., Li X.H., Hu Q.Y. (2020). Treatment of aluminum and fluoride during hydrochloric acid leaching of lepidolite. Hydrometallurgy.

[153] Yan Q., Li X., Wang Z., Wu X., Guo H., Hu Q., Peng W., Wang J. (2012). Extraction of valuable metals from lepidolite. Hydrometallurgy.

[154] Liu G., Zhao Z., Ghahreman A. (2019). Novel approaches for lithium extraction from salt-lake brines: A review. Hydrometallurgy.

[155] Stringfellow W.T., Dobson P.F. (2021). Technology for the Recovery of Lithium from Geothermal Brines. Energies.

[156] Khalil A., Mohammed S., Hashaikeh R., Hilal N. (2022). Lithium recovery from brine: Recent developments and challenges. Desalination.

[157] Zhou R., Wang S., Srinivasakannan C., Li S., Yin S., Zhang L., Jiang X., Zhou G., Zhang N. (2023). Lithium extraction from salt lake brines with high magnesium/lithium ratio: A review. Environ. Chem. Lett..

[158] Flexer V., Baspineiro C.F., Galli C.I. (2018). Lithium recovery from brines: A vital raw material for green energies with a potential environmental impact in its mining and processing. Sci. Total Environ..

[159] Liu X., Zhong M., Chen X., Zhao Z. (2018). Separating lithium and magnesium in brine by aluminum-based materials. Hydrometallurgy.

[160] Xiang W., Liang S., Zhou Z., Qin W., Fei W. (2017). Lithium recovery from salt lake brine by counter-current extraction using tributyl phosphate/FeCl3 in methyl isobutyl ketone. Hydrometallurgy.

[161] Song J., Huang T., Qiu H., Li X.M., He T. (2017). Recovery of lithium from salt lake brine of high Mg/Li ratio using Na [FeCl4.2TBP] as extractant: Thermodynamics, kinetics and processes. Hydrometallurgy.

[162] Yang F., Chen S., Shi C., Xue F., Zhang X., Ju S., Xing W. (2018). A Facile Synthesis of Hexagonal Spinel λ-MnO_2_ Ion-Sieves for Highly Selective Li+ Adsorption. Processes.

[163] Gao A., Sun Z., Li S., Hou X., Li H., Wu Q., Xi X. (2018). The mechanism of manganese dissolution on Li1.6Mn1.6O4 ion sieves with HCl. Dalton Trans..

[164] Wang S., Chen X., Zhang Y., Zhang Y., Zheng S. (2018). Lithium adsorption from brine by iron-doped titanium lithium ion sieves. Particuology.

[165] Kim S., Lee J., Soo J., Jo K., Kim S., Sung Y., Yoon J. (2015). Lithium recovery from brine using a β-MnO_2_/activated carbon hybrid supercapacitor system. Chemosphere.

[166] Lawagon C.P., Nisola G.M., Cuevas R.A.I., Kim H., Lee S.P., Chung W.J. (2018). Li_1−x_Ni_0.33_Co_1/3_Mn_1/3_O_2_/Ag for electrochemical lithium recovery from brine. Chem. Eng. J..

[167] He L., Xu W., Song Y., Luo Y., Liu X., Zhao Z. (2018). New insights into the application of lithium-ion battery materials: Selective extraction of lithium from brines via a rocking-chair lithium-ion battery system. Glob. Chall..

[168] Atta Mends E.L., Chu P. (2023). Lithium extraction from unconventional aqueous resources—A review on recent technological development for seawater and geothermal brines. J. Environ. Chem. Eng..

[169] Kumar A., Fukuda H., Alan Hatton T., Lienhard V. (2019). Lithium Recovery from Oil and Gas Produced Water: A Need for a Growing Energy Industry. ACS Energy Lett..

[170] Zhang J., Cheng Z., Qin X., Gao X., Wang M., Xian X. (2023). Recent advances in lithium extraction from salt lake brine using coupled and tandem technologies. Desalination.

[171] Miller K.N.A., Thompson K.F., Johnston P., Santillo D. (2018). An overview of seabed mining including the current state of development, environmental impacts, and knowledge gaps. Front. Mar. Sci..

[172] Hein J., Mizell K., Koschinsky A., Conrad T. (2013). Deep-ocean mineral deposits as a source of critical metals for high- and green-technology applications: Comparison with land-based resources. Ore Geol. Rev..

[173] Yang S., Zhang F., Ding H., He P., Zhou H. (2018). Lithium Metal Extraction from Seawater. Joule.

[174] Murphy O., Haji M.N. (2022). A review of technologies for direct lithium extraction from low Li+ concentration aqueous solutions. Front. Chem. Eng..

[175] (2020). European Consortium Launches Blue Nodules Project. Press Release. https://www.mining-technology.com/contractors/data//pressreleases/pressblue-nodules-project/.

[176] Brooks C.S. (2018). Metal Recovery from Industrial Waste.

[177] Cui F., Wang G., Yu D., Gan X., Tian Q., Guo X. (2020). Towards “zero waste” extraction of nickel from scrap nickel-based superalloy using magnesium. J. Clean. Prod..

[178] Kim M.S., Lee J.C., Park H.S., Jun M.J., Kim B.S. (2018). A multistep leaching of nickel-based superalloy scrap for selective dissolution of its constituent metals in hydrochloric acid solutions. Hydrometallurgy.

[179] Mamo S.K., Elie M., Baron M.G., Simons A.M., Gonzalez-Rodriguez J. (2019). Leaching kinetics, separation, and recovery of rhenium and component metals from CMSX-4 superalloys using hydrometallurgical processes. Separ. Purif. Technol..

[180] Zhou X., Chen Y., Yin J., Xia W., Yuan X., Xiang X. (2018). Leaching kinetics of cobalt from the scraps of spent aerospace magnetic materials. Waste Manag..

[181] Lupi C., Pilone D. (2002). Ni–MH spent batteries: A raw material to produce Ni–Co alloys. Waste Manag..

[182] Zhang X., Li L., Fan E., Xue Q., Bian Y., Wu F., Chen R. (2018). Toward sustainable and systematic recycling of spent rechargeable batteries. Chem. Soc. Rev..

[183] Baum Z.J., Bird R.E., Yu X., Ma J. (2022). Lithium-Ion Battery Recycling─Overview of Techniques and Trends. ACS Energy Lett..

[184] Latini D., Vaccari M., Lagnoni M., Orefice M., Mathieux F., Huisman J., Tognotti L., Bertei A. (2022). A comprehensive review and classification of unit operations with assessment of outputs quality in lithium-ion battery recycling. J. Power Sources.

[185] Niu B., Xu Z., Xiao J., Qin Y. (2023). Recycling Hazardous and Valuable Electrolyte in Spent Lithium-Ion Batteries: Urgency, Progress, Challenge, and Viable Approach. Chem. Rev..

[186] Mazurek K., Weidner E., Drużyński S., Ciesielczyk F., Kiełkowska U., Wróbel-Kaszanek A., Jesionowski T. (2020). Lanthanum enriched TiO_2_-ZrO_2_ hybrid material with tailored physicochemical properties dedicated to separation of lithium and cobalt(II) raising from the hydrometallurgical stage of the recycling process of lithium-ion batteries. Hydrometallurgy.

[187] Takano M., Asano S., Goto M. (2022). Recovery of nickel, cobalt and rare-earth elements from spent nickel–metal-hydride battery: Laboratory tests and pilot trials. Hydrometallurgy.

[188] Yu W., Guo Y., Shang Z., Zhang Y., Xu S. (2022). A review on comprehensive recycling of spent power lithium-ion battery in China. eTransportation.

[189] Brückner L., Frank J., Elwert T. (2020). Industrial Recycling of Lithium-Ion Batteries—A Critical Review of Metallurgical Process Routes. Metals.

[190] Bruno M., Fiore S. (2023). Material Flow Analysis of Lithium-Ion Battery Recycling in Europe: Environmental and Economic Implications. Batteries.

[191] Meng K., Xu G., Peng X., Youcef-Toumi K., Li J. (2022). Intelligent disassembly of electric-vehicle batteries: A forward-looking overview. Resour. Conserv. Recycl..

[192] Zorn M., Ionescu C., Klohs D., Zähl K., Kisseler N., Daldrup A., Hams S., Zheng Y., Offermanns C., Flamme S. (2022). An Approach for Automated Disassembly of Lithium-Ion Battery Packs and High-Quality Recycling Using ComputerVision, Labeling, and Material Characterization. Recycling.

[193] Ueda T., Koyanaka S., Oki T. (2024). In-line sorting system with battery detection capabilities in e-waste using combination of X-ray transmission scanning and deep learning. Resources. Conserv. Recycl..

[194] Dunn J., Kendall A., Slattery M. (2022). Electric vehicle lithium-ion battery recycled content standards for the US—Targets, costs, and environmental impacts. Resour. Conserv. Recycl..

[195] Gonzales-Calienes G., Kannangara M., Bensebaa F. (2023). Economic and Environmental Viability of Lithium-Ion Battery Recycling—Case Study in Two Canadian Regions with Different Energy Mixes. Batteries.

[196] Kala S., Mishra A. (2021). Battery recycling opportunity and challenges in India. Mater. Today Proc..

[197] Gericke M., Nyanjowa W., Robertson S. (2021). Technology Landscape Report and Business Case for the Recycling of Li-Ion Batteries in South Africa.

[198] Hernández L., Hilbert I., Castillero L.G., Manhart A., García D., Nkongdem B., Dumitrescu R., Sucre C.G., Herrera C.F. (2024). Recycling and Reuse of Lithium Batteries in Latin America and the Caribbean Analytical Review of Global and Regional Practices.

[199] Paulikas D., Katona S., Ilves E., Ali S.H. (2020). Life cycle climate change impacts of producing battery metals from land ores versus deep-sea polymetallic nodules. J. Clean. Prod..

[200] Agboola O., Babatunde D.E., Fayomi O.S.I., Sadiku E.R., Popoola P., Moropeng L., Yahaya A., Mamudu O.A. (2020). A review on the impact of mining operation: Monitoring, assessment and management. Results Eng..

[201] Wei W., Samuelsson P.B., Tilliander A., Gyllenram R., Jonsson P.G. (2020). Energy Consumption and Greenhouse Gas Emissions of Nickel Products. Energies.

[202] Sovacool B.K. (2021). When subterranean slavery supports sustainability transitions? power, patriarchy, and child labor in artisanal Congolese cobalt mining. Extr. Ind. Soc..

[203] Chordia M., Wickerts S., Nordelöf A., Arvidsson R. (2022). Life cycle environmental impacts of current and future battery-grade lithium supply from brine and spodumene. Resour. Conserv. Recycl..

[204] Vera M.L., Torres W.R., Galli C.I., Chagnes A., Flexer V. (2023). Environmental impact of direct lithium extraction from brines. Nat. Rev. Earth Environ..

[205] Sharma S.S., Manthiram A. (2020). Towards more environmentally and socially responsible batteries. Energy Environ. Sci..

